# Healthcare Inequities in Access to Regular Healthcare Providers Among Canadian Adults: Findings From the 2022 Canadian Community Health Survey

**DOI:** 10.7759/cureus.114786

**Published:** 2026-08-19

**Authors:** Oluwagbeminiyi Adepeko, Marvellous O Sowunmi, Akinyele Oladimeji, Adebola A Adeleye, Victor Grace T Green, Adedamola T Ogundipe, Erhiawarie A Eferakeya, Fatima Z Abdulrahman, Azeberoje Osueni

**Affiliations:** 1 Mental Health, Obafemi Awolowo University, Ile-Ife, NGA; 2 Faculty of Medicine, Kharkiv National Medical University, Kharkiv, UKR; 3 Family Medicine, Alberta Health Services, Edmonton, CAN; 4 School of Public Health Sciences, University of Waterloo, Waterloo, CAN; 5 General Medicine, Ivan Horbachevsky Ternopil National Medical University, Ternopil, UKR; 6 Data Science, Pan-Atlantic University, Lekki, NGA; 7 Faculty of Dentistry, University of Lagos, Lagos, NGA; 8 Family Medicine, Ashmed Specialist Hospital, Abuja, NGA; 9 General Medicine, Aminu Kano Teaching Hospital, Kano, NGA; 10 Gastroenterology and Hepatology, Lagos University Teaching Hospital, Surulere, NGA

**Keywords:** canada, canadian community health survey, chronic disease, healthcare access, healthcare inequities, primary care utilization

## Abstract

Background: Healthcare inequities in access to primary care services remain a significant public health concern in Canada. Differences in having a regular healthcare provider may affect continuity of care, chronic disease management, and preventive health service utilization among Canadian adults.

Objective: To evaluate demographic, socioeconomic, geographic, and health-related factors associated with having a regular healthcare provider among Canadian adults using data from the Canadian Community Health Survey 2022.

Methods: This cross-sectional study used data from the 2022 Canadian Community Health Survey, which is representative of the non-institutionalized civilian Canadian adult population, excluding individuals living on indigenous reserves and settlements, members of the Canadian Armed Forces, institutionalized populations, and residents of certain remote regions. Adults aged 18 years and older were included in the analysis. Following complete-case analysis, the final analytic sample consisted of 52,518 respondents representing 26,959,637 non-institutionalized civilian Canadian adults.

Results: Among Canadian adults, 23,148,755 reported having a regular healthcare provider, while 3,810,881 reported no regular healthcare provider. Female adults had higher odds of having a regular healthcare provider compared with males (adjusted odds ratio (aOR) 2.11, 95% confidence interval (CI) 1.90-2.34). Adults aged 65 years and older had greater odds of reporting a regular healthcare provider than adults aged 18-34 years (aOR 3.16, 95% CI 2.73-3.67). Higher income quintiles were also associated with higher odds of having a regular healthcare provider. Diabetes, hypertension, overweight or obesity, and fair or poor perceived health were likewise associated with higher odds of having a regular healthcare provider.

Conclusion: Healthcare inequities in having a regular healthcare provider persist among Canadian adults and vary according to demographic, socioeconomic, and health-related characteristics. These findings highlight important differences in regular healthcare provider status across population groups in Canada. Future research should examine structural and healthcare system factors influencing equitable access to regular healthcare providers.

## Introduction

Global health inequities continue to be a major public concern as it influences factors like access to quality healthcare, quality of care given, and overall population health outcomes [1]. Primary healthcare is the foundation for an effective healthcare system by providing a full range of prevention, early detection, management of chronic disease, health promotion (preventive), and referral to patients for specialty care [2,3]. Therefore, equitable access to primary care is a key component of improving population health, reducing avoidable hospital admissions for primary care-related diseases, and decreasing health disparities amongst various social groups [4]. Although Canada has a universally funded healthcare system, there is still evidence demonstrating inequities in access to regular primary care providers among adults based on socioeconomic status, place of residence, ethnicity/race, level of education, gender, age, and chronic health issues [5,6].

Canada has a publicly funded healthcare system that is designed to provide universal access to medically necessary healthcare services without requiring a person to pay out of their own pocket [7]. The Canada Health Act includes two main principles: access and universality. These principles are intended to enhance equity across the healthcare system in Canada [8].

Primary care access is impacted by numerous factors that are regarded as social determinants of health; these determinants are defined as the conditions in which we live, work, and play and typically include income, employment, education, housing, and social support systems [9,10]. Individuals who are at the bottom of the socioeconomic spectrum tend to experience poorer health while also having greater challenges accessing primary care services [11]. Several studies have illustrated that economically disadvantaged adults in Canada are less likely to have a family physician (or access to one) than their wealthier counterparts, and so have a higher likelihood of getting most of their healthcare needs met from an emergency department instead of from their family physician [12].

Other demographic factors, such as age, sex, race, and immigration status, also influence access to regular primary care providers among Canadian adults. Older adults and those with chronic illnesses typically require more frequent healthcare service visits [13]. In addition, due to various barriers, older adults and those with chronic illnesses may have difficulty accessing appropriate healthcare services in a timely manner [14]. In Canada, both indigenous people and newcomers from other countries continue to experience significantly poorer health status and are also faced with barriers to accessing culturally safe healthcare services as a result of the effects of a long history of inequality, discrimination, and geographical isolation [15]. Similarly, newcomers and people from racialized groups are likely to experience language barriers, unfamiliarity with the healthcare system, and other cultural barriers that negatively affect their ability to utilize healthcare services [16].

The year 2022 marked a significant period for understanding healthcare utilization in Canada, as the healthcare system and services were continuing to operate in the shadow of the COVID-19 pandemic [17]. COVID-19 negatively affected normal healthcare access, added additional healthcare needs, and exacerbated inequities faced by vulnerable individuals [18]. Healthcare system strain, staffing challenges, and health delivery model shifts (including more virtual care) led to delays in accessing primary healthcare services on the part of many Canadians [19].

The study uses data from the Canadian Community Health Survey (CCHS) 2022. CCHS is a nationally representative survey that collects detailed data about the health status, healthcare utilization, and social determinants of health of Canadians [20]. The objective of the study is to evaluate healthcare inequities in access to a regular healthcare provider among Canadian adults using data from the 2022 CCHS.

## Materials and methods

Study design and data source

This study used a cross-sectional analytical design based on data from the 2022 CCHS, a nationally representative survey conducted annually by Statistics Canada [21]. The survey collects information on health status, healthcare utilization, chronic conditions, and health-related behaviors among Canadians aged 12 years and older living in the 10 provinces and three territories. Individuals living on indigenous reserves and settlements, members of the Canadian Armed Forces, institutionalized populations, and persons residing in certain remote regions were excluded from the survey sampling frame. The CCHS applies a multistage stratified cluster sampling design to produce population-level estimates representative of the Canadian population. Public use microdata files and bootstrap replicate weights provided by Statistics Canada were used for the analysis.

Study population

**Figure 1:**
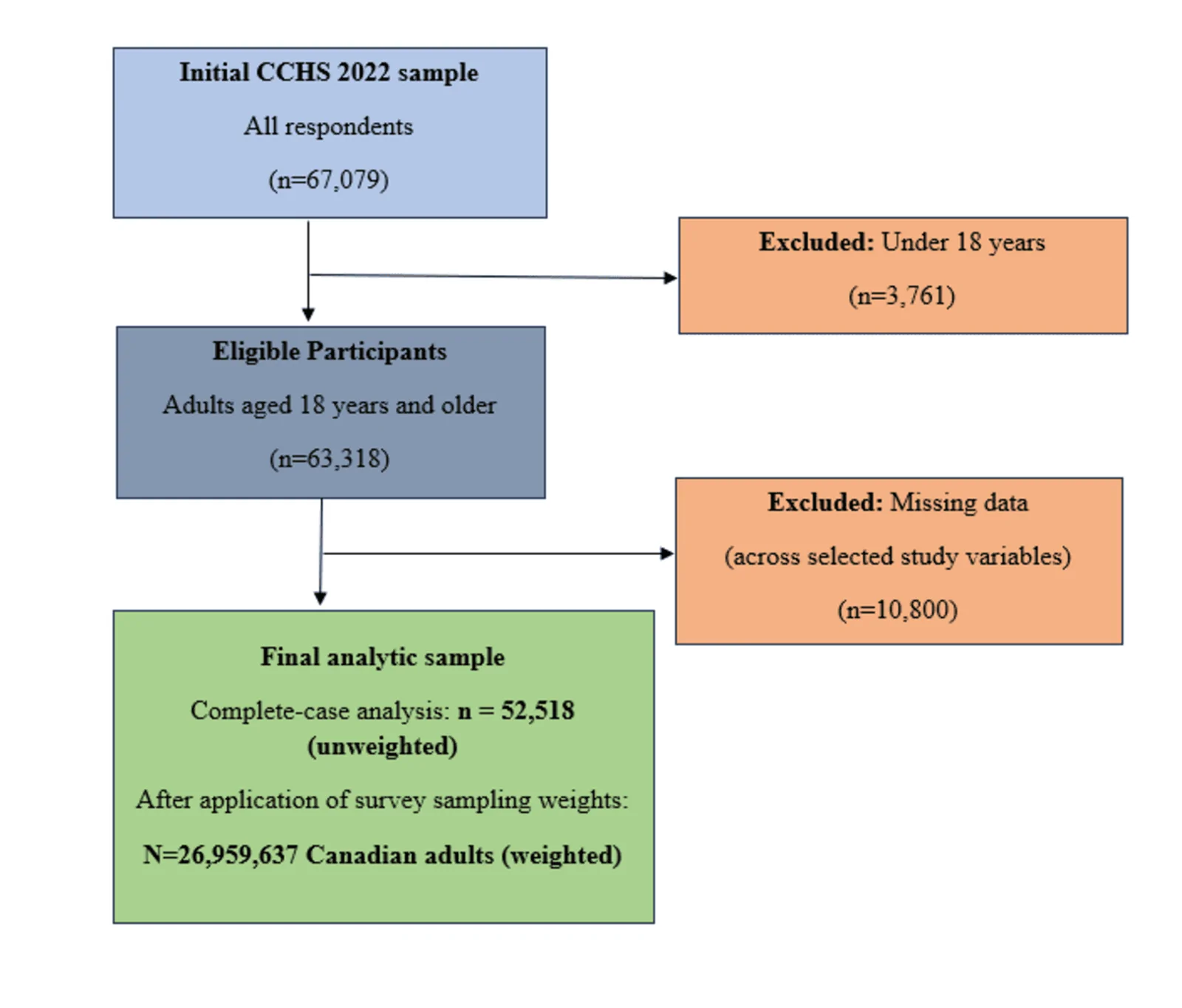
Population Selection Flow Chart (CCHS 2022) CCHS: Canadian Community Health Survey

The study population consisted of Canadian adults aged 18 years and older who participated in the CCHS 2022. The initial survey sample included 67,079 respondents. Restriction to adults aged 18 years and older resulted in the exclusion of 3,761 respondents, leaving 63,318 eligible participants for analysis. Complete-case analysis was applied because the proportion of missing data for individual variables retained in the final analytical model was relatively low and considered unlikely to substantially affect estimate stability. However, the cumulative exclusion of participants with missing data resulted in the removal of 10,800 respondents (approximately 17% of the eligible sample), which may have introduced selection bias if excluded participants differed systematically from those included in the analysis. A total of 10,800 observations were therefore excluded because of missing data across the selected study variables, resulting in a final unweighted analytic sample of 52,518 respondents. After application of survey sampling weights, the final weighted population represented 26,959,637 Canadian adults, as shown in Figure 1.

Variables and measures

The outcome variable was regular healthcare provider status, derived from the CCHS question asking respondents whether they had a regular healthcare provider. Respondents who answered "yes" were subsequently asked to identify the type of healthcare provider they usually consulted. Provider types included a family physician or general practitioner, medical specialist, nurse practitioner, or another healthcare provider. Respondents reporting any of these provider types were classified as having a regular healthcare provider, whereas those who answered "no" were classified as not having a regular healthcare provider. This variable reflects whether respondents reported having a usual healthcare provider and does not measure the frequency of healthcare visits. No minimum visit frequency or specific time frame was applied.

Sociodemographic variables included age group, sex, education level, household income quintile, and province group. Age was categorized into 18-34 years, 35-49 years, 50-64 years, and 65 years and older. Sex was categorized as male or female. Education level was derived from the CCHS education variable and recoded into three categories: less than secondary education, secondary education, and post-secondary education. Household income was measured using nationally standardized household income quintiles and categorized into the lowest, second, middle, fourth, and highest quintiles. Geographic region was derived from the province or territory of residence variable and categorized as Atlantic Canada (Newfoundland and Labrador, Prince Edward Island, Nova Scotia, and New Brunswick), Quebec, Ontario, the Prairies (Manitoba and Saskatchewan), Alberta, British Columbia, and the Territories (Yukon, Northwest Territories, and Nunavut). Provinces were grouped to reduce the number of geographic categories while maintaining meaningful regional comparisons. Alberta was retained as a separate category because of its larger population and sample size, whereas Manitoba and Saskatchewan were combined as the Prairies.

Clinical and behavioral variables included body mass index (BMI) classification, smoking status, alcohol consumption, diabetes, hypertension, and perceived health status. BMI classification was based on the CCHS BMI variable and recoded into two categories: underweight or normal weight and overweight or obese (class I-III). Smoking status was categorized as current smoker, former smoker, or never smoker using the standard CCHS classifications. Alcohol consumption was categorized as regular drinker, occasional drinker, or non-drinker according to the CCHS variable definitions. Diabetes and hypertension were categorized as yes or no based on self-reported physician diagnosis. Perceived health status was categorized as excellent or very good, good, and fair or poor. Cardiovascular disease was initially considered for inclusion in the analysis but was excluded because of substantial missing data that could have significantly reduced the analytical sample size under the complete-case analysis approach. Employment status was also excluded from the final analysis because the variable was collected only for respondents aged 18-64 years, resulting in a high proportion of valid skip responses among older adults.

Missing data

Missing data were assessed for all study variables before analysis. The proportion of missing data was 3.33% for education level, 5.16% for BMI classification, 0.39% for alcohol consumption, 0.46% for regular healthcare provider status, 1.30% for diabetes, 0.78% for hypertension, 3.26% for smoking status, and 4.27% for income quintile. Cardiovascular disease had 18.43% missing data and was excluded from the final analytical model because of concerns regarding sample reduction and potential instability of regression estimates under complete-case analysis. Complete-case analysis was subsequently applied to the remaining variables included in the final regression model.

Statistical analysis

All analyses accounted for the complex survey design of the CCHS using survey sampling weights and bootstrap replicate weights supplied by Statistics Canada. Descriptive characteristics were summarized using survey-weighted counts and percentages. Formal hypothesis tests and p-values were not presented for these descriptive comparisons because the purpose was to describe the distribution of participant characteristics rather than to conduct unadjusted inferential comparisons. Bootstrap replicate weights were used, where applicable, to obtain variance estimates that accounted for the complex survey design.

Multivariable survey-weighted logistic regression analysis was performed to examine factors associated with having a regular healthcare provider among Canadian adults. Adjusted odds ratios and 95% confidence intervals were estimated using bootstrap variance estimation procedures. Variables included in the regression model were sex, age group, education level, income quintile, province group, BMI classification, smoking status, alcohol consumption, diabetes, hypertension, and perceived health status. Statistical significance was assessed at p < 0.05.

Multicollinearity among the independent variables was assessed using variance inflation factors (VIFs). VIF values ranged from 1.05 to 3.37, with a mean VIF of 1.92, indicating no evidence of problematic multicollinearity among the variables included in the final model. A formal goodness-of-fit test for the survey-weighted logistic regression model, such as the Archer-Lemeshow test, was not performed. All statistical analyses were conducted using Stata version 18 (StataCorp LLC, College Station, TX, USA) [22].

Ethical considerations

This study was a secondary analysis of anonymized data from the 2022 CCHS public use microdata file. The dataset contains no personally identifiable information, and no direct participant contact occurred. As a secondary analysis of publicly available anonymized data, ethics approval and additional informed consent were not required.

## Results

Table 1 presents the sociodemographic and clinical characteristics of Canadian adults according to regular healthcare provider status in the CCHS 2022.

**Table 1:** Sociodemographic and Clinical Characteristics of Canadian Adults by Regular Healthcare Provider Status, CCHS 2022 (N=26,959,637; n=52,518) Values are presented as weighted counts and weighted column percentages derived from the Canadian Community Health Survey (CCHS) 2022. Values are presented as survey-weighted counts and column percentages. Formal hypothesis tests were not conducted because Table 1 was intended to provide descriptive, rather than inferential, comparisons. All estimates accounted for the complex survey design using sampling weights and bootstrap replicate weights. All analyses were performed using Stata version 18 (StataCorp LLC, College Station, TX, USA) [22].

Variable	No Regular Provider (N=3,810,881)	Has Regular Provider (N=23,148,755)
Sex, n (%)		
Male	2,473,004 (64.89)	11,093,846 (47.92)
Female	1,337,877 (35.11)	12,054,909 (52.08)
Age group, n (%)		
18-34 years	1,685,837 (44.24)	5,644,634 (24.38)
35-49 years	958,368 (25.15)	5,672,011 (24.50)
50-64 years	719,438 (18.88)	6,039,197 (26.09)
≥65 years	447,238 (11.73)	5,792,913 (25.03)
Education level, n (%)		
Less than secondary	156,121 (4.10)	1,027,435 (4.44)
Secondary school	537,564 (14.10)	3,531,347 (15.26)
Postsecondary	3,117,196 (81.80)	18,589,973 (80.30)
Income quintile, n (%)		
Lowest quintile	913,736 (23.97)	4,251,859 (18.37)
Second quintile	696,483 (18.28)	4,573,597 (19.76)
Middle quintile	804,570 (21.11)	4,652,636 (20.10)
Fourth quintile	700,313 (18.38)	4,814,044 (20.80)
Highest quintile	695,779 (18.26)	4,856,619 (20.97)
Province group, n (%)		
Atlantic Canada	251,491 (6.60)	1,460,966 (6.31)
Quebec	1,317,714 (34.58)	4,858,030 (20.99)
Ontario	1,079,796 (28.33)	9,560,859 (41.30)
Prairies	209,157 (5.49)	1,362,376 (5.89)
Alberta	334,894 (8.79)	2,777,721 (12.00)
British Columbia	617,829 (16.21)	3,128,803 (13.51)
BMI category, n (%)		
Underweight/normal weight	1,538,587 (40.37)	7,989,641 (34.51)
Overweight/obese	2,272,294 (59.63)	15,159,114 (65.49)
Smoking status, n (%)		
Current smoker	585,244 (15.36)	2,372,140 (10.25)
Former smoker	689,599 (18.10)	5,565,099 (24.04)
Never smoker	2,536,038 (66.54)	15,211,516 (65.71)
Alcohol consumption, n (%)		
Regular drinker	2,331,154 (61.17)	13,384,789 (57.82)
Occasional drinker	652,745 (17.13)	4,357,640 (18.82)
Non-drinker	826,982 (21.70)	5,406,326 (23.35)
Diabetes, n (%)		
Yes	96,758 (2.54)	2,008,799 (8.68)
No	3,714,123 (97.46)	21,139,956 (91.32)
Hypertension, n (%)		
Yes	361,809 (9.49)	5,089,671 (21.99)
No	3,449,072 (90.51)	18,059,084 (78.01)
Perceived health, n (%)		
Excellent/very good	2,353,068 (61.74)	12,431,618 (53.70)
Good	1,075,390 (28.22)	7,396,024 (31.95)
Fair/poor	382,423 (10.04)	3,321,113 (14.35)

Among Canadian adults included in the analysis, 23,148,755 individuals reported having a regular healthcare provider, while 3,810,881 reported having no regular healthcare provider. Females represented a larger proportion of individuals with a regular healthcare provider, 12,054,909 (52.08%), compared with males, 11,093,846 (47.92%). In contrast, males accounted for most individuals without a regular healthcare provider, 2,473,004 (64.89%).

The proportion of adults with a regular healthcare provider increased progressively across age groups. Adults aged 65 years and older accounted for 5,792,913 (25.03%) of those with a regular healthcare provider, compared with 447,238 (11.73%) among those without a regular healthcare provider. Adults aged 18 to 34 years represented the largest proportion of individuals without a regular healthcare provider, 1,685,837 (44.24%).

Most participants in both groups had postsecondary education. Among those with a regular healthcare provider, 18,589,973 (80.30%) had postsecondary education, while 3,117,196 (81.80%) of those without a regular healthcare provider also had postsecondary education. Higher income quintiles were more common among adults with a regular healthcare provider. The highest income quintile represented 4,856,619 (20.97%) of individuals with a regular healthcare provider, compared with 695,779 (18.26%) among those without a regular healthcare provider.

Regional differences were also observed. Ontario accounted for the largest proportion of adults with a regular healthcare provider, 9,560,859 (41.30%), whereas Quebec represented the largest proportion among adults without a regular healthcare provider, 1,317,714 (34.58%).

Adults classified as overweight or obese represented 15,159,114 (65.49%) of individuals with a regular healthcare provider and 2,272,294 (59.63%) of those without a regular healthcare provider. Never smokers accounted for most participants in both groups. Diabetes and hypertension were more common among adults with a regular healthcare provider. Diabetes was reported among 2,008,799 (8.68%) adults with a regular healthcare provider compared with 96,758 (2.54%) among those without a regular healthcare provider. Similarly, hypertension was reported among 5,089,671 (21.99%) adults with a regular healthcare provider and 361,809 (9.49%) adults without a regular healthcare provider.

Perceived health status differed across provider groups. Fair or poor perceived health was more frequent among adults with a regular healthcare provider, 3,321,113 (14.35%), compared with 382,423 (10.04%) among adults without a regular healthcare provider.

Table 2 presents the multivariable survey-weighted logistic regression analysis examining factors associated with having a regular healthcare provider among Canadian adults.

**Table 2:** Multivariable Survey-Weighted Logistic Regression Analysis of Factors Associated With Having a Regular Healthcare Provider Among Canadian Adults, CCHS 2022 Adjusted odds ratios (ORs) and 95% confidence intervals (CIs) were estimated using survey-weighted logistic regression models that accounted for the complex sampling design of the Canadian Community Health Survey (CCHS) 2022, including bootstrap variance estimation. An asterisk (*) indicates statistical significance at p<0.05. The regression model was adjusted for sex, age group, education level, income quintile, province group, BMI classification, smoking status, alcohol consumption, diabetes, hypertension, and perceived health status. All analyses were performed using Stata version 18 (StataCorp LLC, College Station, TX, US) [22].

Variable	Adjusted OR	95% CI	p-value
Sex			
Female vs male	2.11	1.90-2.34	<0.001*
Age group			
35-49 years vs 18-34 years	1.69	1.48-1.93	<0.001*
50-64 years vs 18-34 years	2.17	1.89-2.50	<0.001*
≥65 years vs 18-34 years	3.16	2.73-3.67	<0.001*
Education level			
Secondary education vs less than secondary	1.07	0.85-1.34	0.553
Post-secondary education vs less than secondary	1.08	0.87-1.34	0.500
Income quintile			
Second income quintile vs lowest income quintile	1.53	1.30-1.80	<0.001*
Middle income quintile vs lowest income quintile	1.33	1.12-1.59	0.001*
Fourth income quintile vs lowest income quintile	1.70	1.43-2.03	<0.001*
Highest income quintile vs lowest income quintile	1.67	1.42-1.98	<0.001*
Province group			
Quebec vs Atlantic Canada	0.68	0.58-0.79	<0.001*
Ontario vs Atlantic Canada	1.75	1.48-2.07	<0.001*
Prairies vs Atlantic Canada	1.28	1.04-1.58	0.018*
Alberta vs Atlantic Canada	1.69	1.41-2.01	<0.001*
British Columbia vs Atlantic Canada	0.93	0.78-1.10	0.381
Body mass index classification			
Overweight/obese vs underweight/normal weight	1.13	1.01-1.27	0.031*
Smoking status			
Former smoker vs current smoker	1.49	1.26-1.77	<0.001*
Never smoker vs current smoker	1.43	1.23-1.65	<0.001*
Alcohol consumption			
Occasional drinker vs regular drinker	1.03	0.90-1.19	0.626
Non-drinker vs regular drinker	0.96	0.83-1.11	0.603
Diabetes			
Yes vs no	2.26	1.77-2.88	<0.001*
Hypertension			
Yes vs no	1.59	1.36-1.86	<0.001*
Perceived health			
Good vs excellent/very good	1.10	0.98-1.24	0.097
Fair/poor vs excellent/very good	1.21	1.03-1.42	0.023*

Female adults had higher odds of having a regular healthcare provider compared with males (adjusted OR 2.11, 95% CI 1.90 to 2.34, p < 0.001). Increasing age was associated with higher odds of having a regular healthcare provider. Adults aged 35 to 49 years had 1.69 times higher odds of having a regular healthcare provider compared with adults aged 18 to 34 years. Adults aged 50 to 64 years had adjusted odds of 2.17, while adults aged 65 years and older had adjusted odds of 3.16.

Education level was not significantly associated with having a regular healthcare provider. Compared with the lowest income quintile, adults in higher income quintiles had significantly higher odds of having a regular healthcare provider. Although the adjusted odds ratio for the fourth income quintile was slightly higher than that for the highest income quintile, the confidence intervals overlapped considerably, suggesting little meaningful difference between these two groups.

Regional differences were identified across province groups. Compared with Atlantic Canada, adults residing in Ontario and Alberta had higher odds of having a regular healthcare provider, while adults residing in Quebec had lower odds. No statistically significant association was observed for British Columbia.

Adults classified as overweight or obese had slightly higher odds of having a regular healthcare provider compared with adults classified as underweight or normal weight (adjusted OR 1.13, 95% CI 1.01 to 1.27, p = 0.031). Former smokers and never smokers had higher odds of having a regular healthcare provider compared with current smokers.

Alcohol consumption was not significantly associated with having a regular healthcare provider. Adults with diabetes had more than two times higher odds of having a regular healthcare provider compared with adults without diabetes (adjusted OR 2.26, 95% CI 1.77 to 2.88, p < 0.001). Hypertension was also significantly associated with having a regular healthcare provider (adjusted OR 1.59, 95% CI 1.36 to 1.86, p < 0.001).

Adults reporting fair or poor perceived health had higher odds of having a regular healthcare provider compared with those reporting excellent or very good perceived health (adjusted OR 1.21, 95% CI 1.03 to 1.42, p = 0.023).

**Figure 2:**
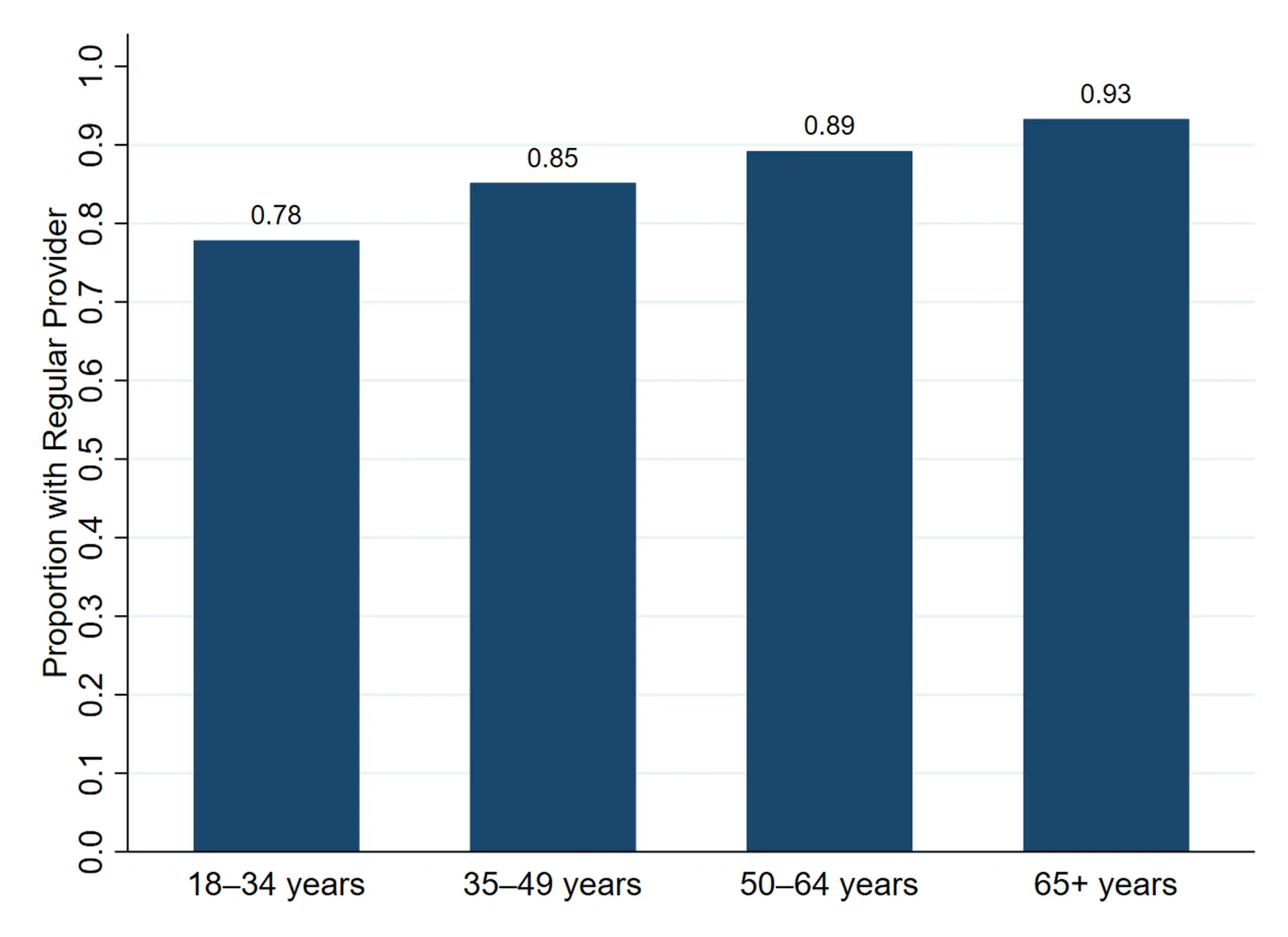
Unweighted Proportion of Respondents Reporting a Regular Healthcare Provider by Age Group

Figure 2 illustrates the proportion of Canadian adults with a regular healthcare provider across age groups.

The proportion of adults with a regular healthcare provider increased progressively across age groups. Adults aged 18 to 34 years had the lowest proportion with a regular healthcare provider at 0.78. The proportion increased to 0.85 among adults aged 35 to 49 years, 0.89 among adults aged 50 to 64 years, and 0.93 among adults aged 65 years and older. The findings indicate a consistent increase in regular healthcare provider utilization with advancing age.

## Discussion

This study examined factors associated with having a regular healthcare provider among Canadian adults using nationally representative data from the CCHS 2022. The findings showed that female sex, older age, higher income, overweight or obesity, diabetes, hypertension, and poorer perceived health were associated with higher odds of having a regular healthcare provider. Regional differences were also observed, particularly among adults residing in Ontario and Alberta, while adults residing in Quebec had lower odds of reporting a regular healthcare provider. Smoking status was also associated with healthcare provider utilization, whereas alcohol consumption and education level were not significantly associated with the outcome after adjustment. These findings are consistent with previous evidence showing that the likelihood of having a regular healthcare provider varies according to demographic, socioeconomic, and health-related factors in Canada [1,4-6]. While some of these differences may reflect inequities in access to regular healthcare providers, others, particularly those related to older age and chronic disease status, are likely to reflect greater healthcare needs and more frequent interaction with the healthcare system.

The observed associations related to age and chronic disease status are clinically important because adults with diabetes and hypertension were more likely to report having a regular healthcare provider. These findings likely reflect increased healthcare needs and ongoing engagement with healthcare services rather than inequitable access alone. Existing evidence indicates that chronic disease prevention and management in Canada depends heavily on continuity of care within primary care settings [2,3]. Adults living with chronic conditions often require regular monitoring, medication management, and follow-up visits, which may increase sustained contact with healthcare providers. Previous Canadian studies have also shown that people with chronic conditions report greater healthcare needs and more frequent interaction with the healthcare system [18]. The higher odds observed among older adults may reflect increasing healthcare utilization with aging, particularly for preventive care, chronic disease management, and medication follow-up [14]. Community-based primary care services are also frequently used among older adults because of mobility limitations and complex health needs [14].

The association between income and having a regular healthcare provider supports previous evidence showing that socioeconomic conditions remain strongly linked to healthcare access in Canada despite universal healthcare coverage [1,4,10]. Adults in higher income quintiles had greater odds of reporting a regular healthcare provider compared with those in the lowest income group. Previous studies have reported that lower socioeconomic status is associated with barriers to healthcare access, avoidable hospitalizations, and delayed care utilization [4,6,12]. Financial limitations may affect transportation, time availability, digital access, and the ability to navigate healthcare systems, even in publicly funded healthcare systems [10]. Studies conducted in Canadian primary care settings have also shown that organizational structures and healthcare delivery models may influence access among socioeconomically disadvantaged populations [11]. Although the adjusted odds ratio for the fourth income quintile was slightly higher than that for the highest income quintile, the confidence intervals overlapped substantially, suggesting little meaningful difference between these upper-income groups.

Regional differences identified in this study are also consistent with growing concerns regarding unequal access to primary care services across Canada [1,9,19]. Adults residing in Ontario and Alberta had higher odds of having a regular healthcare provider, while lower odds were observed in Quebec. Previous reports have highlighted widening disparities in access to primary care across Canadian provinces, partly related to workforce shortages, regional healthcare organization, and primary care capacity pressures [1,19]. Healthcare access challenges became more visible during and after the COVID-19 pandemic, particularly in regions experiencing shortages of family physicians and increased strain on healthcare systems [17,19]. Access to primary care has also been linked to local healthcare infrastructure and the availability of community-based services [9].

The observed association between smoking status and regular healthcare provider use may reflect increased interaction with healthcare services among former smokers and individuals attempting smoking cessation. Preventive counseling and chronic disease screening within primary care settings may contribute to more regular provider contact among these groups [3]. The association between poorer perceived health and higher odds of having a regular healthcare provider may indicate that individuals experiencing poorer health are more likely to seek continuing healthcare services because of ongoing medical concerns or symptom burden. Similar patterns have been reported in studies examining unmet healthcare needs and healthcare utilization among populations with greater health burdens [6,18]. This association likely reflects greater healthcare need and continued engagement with healthcare services rather than differences in access alone.

Current healthcare recommendations in North America strongly emphasize continuous access to primary care, preventive services, and chronic disease management. The United States Preventive Services Task Force recommends regular blood pressure screening, diabetes screening among high-risk adults, smoking cessation counseling, and preventive risk assessment through primary care settings. These recommendations depend heavily on stable access to healthcare providers and continuity of care. The findings of this study underscore the importance of ensuring that Canadian adults have a regular healthcare provider to support preventive care and chronic disease management. Canadian evidence has also emphasized the role of primary care in reducing avoidable hospitalizations and improving management of chronic diseases [2-4].

Strengths and limitations

This study has several strengths and limitations. The use of nationally representative survey data representative of the non-institutionalized Canadian adult population covered by the CCHS sampling frame, together with bootstrap variance estimation, strengthened the generalizability of the findings. The analysis also accounted for the complex survey design.

Several limitations should be considered. The cross-sectional design limits temporal interpretation of associations. All measures were self-reported and may be affected by recall bias or reporting inaccuracies. The outcome measured whether respondents reported having a regular healthcare provider and did not capture the frequency, continuity, or quality of care received. Consequently, some observed associations, particularly those related to older age, diabetes, hypertension, and poorer perceived health, may reflect greater healthcare needs and ongoing interaction with the healthcare system rather than inequitable access alone. Complete-case analysis excluded approximately 17% of eligible respondents and may have introduced selection bias if excluded participants differed systematically from those retained in the analysis. Employment status was excluded because the variable was restricted to adults aged 18 to 64 years, resulting in a high proportion of valid skip responses among older adults. Cardiovascular disease was also excluded because of substantial missing data. In addition, a formal goodness-of-fit assessment for the survey-weighted logistic regression model was not performed. Variables related to healthcare accessibility, physician supply, health insurance supplements, transportation barriers, immigration status, and virtual care access were not available in the data. Future studies should explore longitudinal patterns of having a regular healthcare provider and incorporate broader structural determinants influencing access to healthcare services in Canada.

## Conclusions

This study identified differences in having a regular healthcare provider among Canadian adults using data representative of the non-institutionalized Canadian adult population covered by the CCHS 2022 sampling frame. The likelihood of having a regular healthcare provider differed according to age, sex, household income, chronic disease status, perceived health, and geographic region. The higher likelihood observed among older adults and individuals with chronic conditions or poorer perceived health may reflect greater healthcare needs and more frequent engagement with healthcare services, whereas socioeconomic and regional differences may indicate persistent disparities in access to a regular healthcare provider. These findings suggest that having a regular healthcare provider remains unevenly distributed across the Canadian population. Improving access to regular healthcare providers may strengthen continuity of care and support timely prevention and management of chronic health conditions, particularly among underserved populations. Future research should examine healthcare system and structural factors associated with having a regular healthcare provider in Canada.
